# Incidence trends and relative survival of colorectal neuroendocrine neoplasms: A population‐based study using German cancer registry data

**DOI:** 10.1002/ijc.35372

**Published:** 2025-02-20

**Authors:** Lennart Möller, Andras Szentkirályi, Christine Eisfeld, Ina Wellmann, Franziska Rees, Kevin Claaßen, Florian Oesterling, Hiltraud Kajüter, Andreas Stang

**Affiliations:** ^1^ Cancer Registry of North Rhine Westphalia Bochum Germany; ^2^ Faculty of Medicine University of Duisburg‐Essen Essen Germany; ^3^ Department of Medical Statistics and Epidemiology Medical School Hamburg Hamburg Germany; ^4^ Institute of Medical Informatics, Biometry and Epidemiology University Hospital Essen Essen Germany; ^5^ School of Public Health, Department of Epidemiology Boston University Boston Massachusetts USA

**Keywords:** Germany, incidence, neuroendocrine neoplasms, registries, survival analysis

## Abstract

Neuroendocrine neoplasms (NENs) of the colon and rectum are a heterogeneous group of epithelial neoplasms with neuroendocrine differentiation. They include well‐differentiated neuroendocrine tumors (NETs), poorly differentiated neuroendocrine carcinomas (NECs) and mixed neuroendocrine‐non‐neuroendocrine neoplasms (MiNENs). Our aim was to calculate incidence, incidence trends and relative survival for colonic and rectal NETs, NECs, and MiNENs. We analyzed data covering the entire German population recorded between 2009 and 2021, calculating age‐standardized incidence rates, annual percent changes, and the relative 5‐year survival probability for the calendar period 2017–2021. Our comprehensive analyses included 12,602 NEN cases, with 59% located in the rectum. NECs, MiNENs and tumors with colonic location showed higher stages. We observed an increase in the incidence of NETs, particularly in patients aged <55 years, and in the incidence of MiNENs, and a constant incidence of NECs. The relative five‐year survival was high for rectal NETs (95.9%, 95%‐CI 94.6; 97.1) and colonic NETs (81.4%, 95%‐CI 78.3; 84.5) and low for colonic NECs (20.5%, 95%‐CI 17.6; 23.4) and rectal NECs (19.2%, 95%‐CI 15.7; 22.6). The increase in the incidence of NETs might be partly due to colorectal cancer screening, improved diagnostics, and changes in classification of NETs. We attribute the increase in incidence of MiNENs to the recent introduction of this morphological category. Higher stages at diagnosis, a higher proportion of NECs and higher median age at diagnosis may contribute to the less favorable survival probabilities associated with colonic as opposed to rectal location.

## Graphical abstract

What's new?

This study used data from all 16 German cancer registries for colorectal neuroendocrine neoplasms to calculate incidence and relative survival. The data allowed differentiation by primary site, morphology (well‐differentiated neuroendocrine tumors—NETs, poorly differentiated neuroendocrine carcinomas—NECs and mixed neuroendocrine‐non‐neuroendocrine neoplasms—MiNENs), age, sex, and staging. An increase in NETs and MiNENs incidence and a constant NECs incidence were observed. Survival was high for NETs and low for NECs and MiNENs.

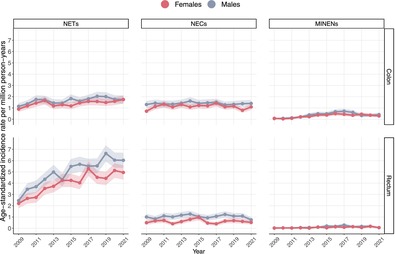

## 1 INTRODUCTION

Neuroendocrine neoplasms (NENs) are a rare and heterogeneous group of tumors that can arise in almost any organ and tissue and are characterized by the presence of neurosecretory granules.^1^ The most common sites for NENs are the digestive tract and the lungs.^2^, ^3^ NENs of the colon and rectum are epithelial neoplasms with neuroendocrine differentiation. They can be distinguished into well‐differentiated neuroendocrine tumors (NETs), poorly differentiated neuroendocrine carcinomas (NECs) and mixed neuroendocrine‐non‐neuroendocrine neoplasms (MiNENs) based on morphology and markers of proliferation such as Ki‐67 index and/or mitotic rate.^4^, ^5^

Due to different natural histories of NENs in colon versus rectum, they are classified as distinct primary sites.^6^ The clinical manifestation of NENs also differ between colonic and rectal sites; NENs of the colon are more frequently detected later at higher stages, while NENs of the rectum tend to be more frequently detected at lower stages and also tend to be more frequently low‐grade at diagnosis.^7^ Most NETs are clinically silent or are associated with unspecific general symptoms, bleeding, and pain.^4^

NENs account for <1% of all colorectal tumors,^8^ but their incidence has been increasing since the 1970s in countries reporting cancer incidence data.^8^, ^9^ The incidence varies by geographic location and ethnicity. Higher rates were reported in North America and Europe, as well as among individuals of Asian and African American descent.^2^, ^10^ The geographical differences are partly related to different diagnostic routines, the availability of colorectal cancer screening and ethnic differences.^10^

The etiology of NENs is unclear and there is little information on risk factors.^11^ A family history of cancer, smoking, alcohol and obesity are considered to be risk factors of NENs.^11^ The reasons of the increase in the incidence of NENs are not well understood. Better understanding of the clinical features and symptoms of NENs leading to more accurate diagnostics,^12^ the widespread use of endoscopy, colorectal cancer screening and imaging methods have been identified as the main causes for the increase in the incidence of NENs in some countries.^10^, ^12^

Colonoscopy enables detection and removal of asymptomatic tumors as well as precursor lesions. According to the screening recommendations of the German statutory health insurances, since 2002, persons aged between 50 and 54 years have been entitled to one fecal occult blood test (FOBT) every year, and above 55 years of age to two colonoscopies at an interval of at least 10 years. In 2019, the lower age limit for screening colonoscopy in men has been reduced to 50 years of age. Nevertheless, the use of screening colonoscopy did not increase in 2020 and 2021 due to the SARS‐CoV‐2 pandemic.^13^

NETs have a better prognosis than NECs and MiNENs and survival with rectal NENs is higher than with colonic NENs.^14^, ^15^, ^16^ Survival probability for NENs has been increasing over time,^2^, ^15^ most likely due to more efficient treatment options^17^ and earlier detection.^2^ For small NETs (10 mm or less) endoscopic resection is a common treatment option, while surgical excision is the main treatment for local or locoregional NETs, NECs and MiNENs in combination with adjuvant chemotherapy for the latter two.^6^, ^18^, ^19^, ^20^, ^21^ Patients with metastatic NEC or MiNEN are routinely treated with chemotherapy, even though there is no established treatment paradigm and the best therapeutic approach for metastatic NETs is unknown.^18^, ^19^, ^21^

The epidemiology of colorectal NENs within the German population has not been studied yet.^22^, ^23^ Furthermore, there are no studies on relative survival and the available analyses do not distinguish between primary site and NET/NEC/MiNEN nor between age and tumor stage. Our aim was therefore to characterize incidence rates, incidence time trends and relative survival of colorectal NECs, NETs, and MiNENs in Germany stratified by primary site, sex, age groups, and stages.

## 2 MATERIALS AND METHODS

This study is based on data transferred to the German Centre for Cancer Registry Data (ZfKD) by the federal population‐based cancer registries.^24^ Cancer reporting in Germany is mandatory. We used the pooled dataset from the ZfKD (Version October 2024) including data from all 16 German states covering a population of 84 million residents.

We included all malignant neuroendocrine neoplasms of the colon and rectum with a date of diagnosis between 2009 and 2021. Tumors of the appendix (C18.1) were excluded since they differ from other colorectal cancers in terms of their biology and etiology.^25^, ^26^ Based on morphological or clinical reports, tumors were classified and analyzed according to the International Classification of Diseases for Oncology (ICD‐O‐3) by primary site as colon (C18) and rectum (C19–C20), by morphology, and grading (G1‐G3) as NET (8152 [L‐cell tumor], 8240 [Neuroendocrine tumor, not otherwise specified], 8241 [Enterochromaffin cell carcinoid], 8246 [Neuroendocrine carcinoma, NOS] if tumor grade was 1 or 2, 8249 [Neuroendocrine tumor, grade 2]), NEC (8013 [Large cell neuroendocrine carcinoma], 8041 [Small cell carcinoma, NOS], 8246 [Neuroendocrine carcinoma, NOS] for grade 3 tumors) and MiNEN (8154 [Mixed neuroendocrine non‐neuroendocrine neoplasm (MiNEN)], 8244 [Mixed adenoneuroendocrine carcinoma], 8245 [Adenocarcinoid tumor]).^27^ We grouped NETs, NECs and MiNENs according to ICD‐O‐3,^27^ WHO Classification of Tumors^4^, ^28^ and previous literature.^3^, ^29^ In addition, the analyses were stratified by sex and age (<55 years vs. ≥55 years) and Union for International Cancer Control (UICC) stage at the time of diagnosis.^30^

### 2.1 Statistical Methods

We calculated age‐standardized incidence rates using the old European standard population,^31^ the world standard population and the US 2000 standard population. Temporal trends were described by estimated annual percentage changes (APC). The APC was estimated by fitting a regression line to the natural logarithm of the annual rates using the calendar years as predictor variables: *Y* = *a* + *bx*, where *Y* = ln(rate) and *x* is the calendar year, and APC = 100 × (*e* ^*b*^ − 1).

We calculated the relative 5‐year survival (RS) for the calendar period 2017–2021. The RS is a computational method for determining cancer‐specific survival probability defined by the ratio of the observed survival of patients and the expected survival of the general population of the same age, sex, and calendar period.^32^ The expected survival was calculated using the life tables for Germany with the Ederer II method.^33^, ^34^ RS was estimated using the period approach^35^ that provides more recent survival estimates than the traditional cohort approach, by using survival information only from the last calendar period for which mortality data are available.^36^ Death‐certificate‐only (DCO) cases were excluded from the survival analysis. The calculations were performed with R 4.3.2,^37^ the RS was estimated with the periodR package, version 1.0‐6^38^ and the Figure 2 was created with SAS software 9.4 for windows (SAS Institute, Cary, NC).

We performed multiple imputation to handle the high proportion of missing stage (UICC) values. Statistical details of the multiple imputation can be found in the Data S1.

## 3 RESULTS

Table 1 shows the characteristics of the study population with a total of 12,602 cases between 2009 and 2021. Ninety‐seven percent of all tumors were morphologically verified. The distribution of morphologies and grading is shown in Table S1. NETs comprised 45% of NENs in the colon and 80% of NENs in the rectum. Rectal NETs and NECs had a lower median age of diagnosis (59 and 68 years) compared to colonic NETs and NECs (66 and 72 years), while colonic and rectal MiNENs had the highest age of diagnosis (73 and 72). For a comprehensive depiction of the age distribution based on age‐specific incidence rates, please refer to Figure S1. Among the 5702 patients with recorded UICC stage information, 83% of rectal NETs were of UICC stages I–II and 69% of colonic NECs had UICC stage IV. More than one third of colonic NETs vs. 10% of rectal NETs were metastasized at diagnosis. MiNENs and NECs had higher stages at diagnosis than NETs, and the difference in stages was more pronounced in the rectum. No staging information was available for 37% of colonic NENs and for 67% of rectal NENs. The staging of MiNENs was the least likely to be missing (22% for colon, 36% for rectum). Young age at diagnosis, localization (rectum), morphology (NETs) and vital status (alive) at the end of the observed time period (not shown) were predictors of missing UICC data and were therefore used for multiple imputation. The UICC stages were shifted toward a more favorable distribution following the imputation as compared to the original complete case dataset (Figure S2).

**TABLE 1:** Characteristics of the study population for colorectal neuroendocrine neoplasms in Germany, 2009–2021.

	Well‐differentiated neuroendocrine tumors (NETs)		Poorly differentiated neuroendocrine carcinomas (NECs)		Mixed neuroendocrine‐non‐neuroendocrine neoplasm (MiNENs)		Neuroendocrine neoplasms (NENs)
	Males	Females	Males	Females	Males	Females	Total
*Colon*							
N (row percentage)	1196 (23)	1114 (22)	1104 (22)	1116 (22)	297 (6)	293 (6)	5120 (100)
Younger than 55 (%)	259 (21.7)	245 (22)	130 (11.8)	94 (8.4)	42 (14.1)	29 (9.9)	799 (16)
Median age at diagnosis with 10th‐ and 90th‐percentile	65 [47–80]	66 [46–82]	70 [53–84]	74 [56–87]	70 [51–83]	76 [55–86]	69 [50–84]
*UICC* (%)							
Non‐missing	615	610	768	753	237	224	3207
I/II	142 (23.1)	121 (19.8)	72 (9.4)	111 (14.7)	24 (10.1)	21 (9.4)	491 (15.3)
III	231 (37.6)	222 (36.4)	128 (16.7)	162 (21.5)	84 (35.4)	66 (29.5)	893 (27.8)
IV	242 (39.3)	267 (43.8)	568 (74)	480 (63.7)	129 (54.4)	137 (61.2)	1823 (56.8)
Missing	581	504	336	363	60	69	1913
*Rectum*							
N (row percentage)	3292 (44)	2716 (36)	796 (11)	520 (7)	96 (1)	62 (1)	7482 (100)
Younger than 55 (%)	1041 (31.6)	931 (34.3)	125 (15.7)	102 (19.6)	12 (12.5)	12 (19.4)	2223 (30)
Median age at diagnosis with 10th‐ and 90th percentile	59 [42–75]	58 [40–76]	68 [51–83]	68 [48–85]	71 [54–85]	72 [47–85]	60 [43–78]
*UICC* (%)							
Non‐missing	861	725	483	310	69	47	2495
I/II	691 (80.3)	621 (85.7)	59 (12.2)	46 (14.8)	8 (11.6)	10 (21.3)	1435 (57.5)
III	52 (6)	46 (6.3)	95 (19.7)	81 (26.1)	18 (26.1)	18 (38.3)	310 (12.4)
IV	118 (13.7)	58 (8)	329 (68.1)	183 (59)	43 (62.3)	19 (40.4)	750 (30.1)
Missing	2431	1991	313	210	27	15	4987

Abbreviation: UICC, stage according to union for international cancer control.

**FIGURE 1:**
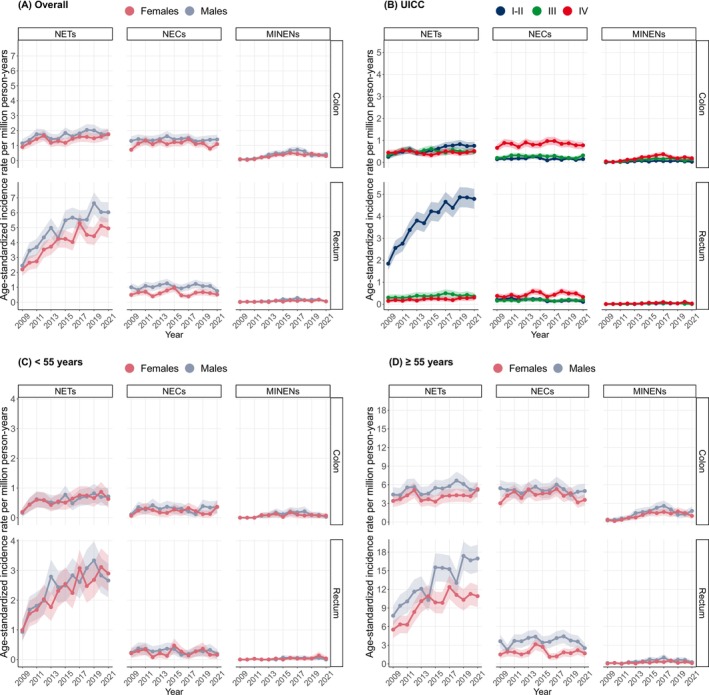
Age‐standardized incidence rates for colorectal neuroendocrine neoplasms, 2009–2021, Germany. NEC, poorly differentiated neuroendocrine carcinoma; NET, well‐differentiated neuroendocrine tumor; MiNEN, mixed neuroendocrine‐non‐neuroendocrine neoplasm; UICC, stage according to union for international cancer control.

Table 2 shows the age‐standardized incidence rates per million person‐years for the latest period from 2019 to 2021 and the APC for the years 2009 to 2021 according to morphology, anatomical site, age, sex, and imputed UICC stages. The incidence of NETs increased within the observed time period in the colon and rectum, in younger and older patients, and in all UICC stages except for UICC IV in colonic NETs. The incidence of NECs overall remained constant, except for a decrease in the incidence of rectal UICC I–II NECs. The incidence of MiNEN increased markedly except for younger patients for colonic MiNENs. Figure 1 shows the age‐standardized incidence rates per million person‐years for the years 2009–2021. Figure S3 shows the age‐standardized incidence rates per million person‐years for the years 2009–2021 stratified by UICC without imputation. Age‐standardized incidence rates (world standard population and US 2000 standard population) are shown in Tables S2 and S3.

**TABLE 2:** Incidence and time trends for colorectal neuroendocrine neoplasms, 2009–2021, Germany.

	Sex	Well‐differentiated neuroendocrine tumors (NETs)				Poorly differentiated neuroendocrine carcinomas (NECs)				Mixed neuroendocrine‐non‐neuroendocrine neoplasm (MiNENs)			
		ASR 2019–2021	95% CI	APC 2009–2021	95% CI	ASR 2019–2021	95% CI	APC 2009–2021	95% CI	ASR 2019–2021	95% CI	APC 2009–2021	95% CI
*Colon*													
Overall	Males	1.85	1.63; 2.06	3.06	1.26; 4.89	1.37	1.2; 1.55	0.02	−0.94; 0.99	0.36	0.28; 0.45	16.9	7.47; 27.16
	Females	1.61	1.41; 1.81	3.44	1.4; 5.52	1.01	0.87; 1.15	0.3	−2.59; 3.27	0.36	0.28; 0.45	17.64	8.42; 27.64
<55 years	Males	0.75	0.57; 0.92	7.49	2.65; 12.56	0.36	0.24; 0.49	2.07	−4.26; 8.82	0.07	0.02; 0.12	−5.32	−17.52; 8.68
	Females	0.72	0.55; 0.9	7.33	3.27; 11.55	0.20	0.11; 0.3	2.4	−4.74; 10.07	0.08	0.02; 0.13	−1.71	−12.47; 10.37
≥55 years	Males	5.49	4.77; 6.2	1.91	0.18; 3.67	4.76	4.11; 5.41	−0.47	−1.68; 0.77	1.41	1.05; 1.77	15.35	7.23; 24.08
	Females	4.59	3.97; 5.22	1.79	−0.19; 3.82	3.78	3.24; 4.32	−0.22	−2.9; 2.52	1.35	1.03; 1.68	16.06	8.2; 24.49
*UICC*													
I/II	Both	0.77	0.67; 0.88	7.99	4.46; 11.64	0.15	0.11; 0.19	−1.91	−7.2; 3.69	0.05	0.02; 0.07	10.53	−1.7; 24.29
III	Both	0.49	0.4; 0.58	1.51	−1.81; 4.94	0.23	0.18; 0.29	−0.52	−4.56; 3.69	0.12	0.08; 0.15	16.26	5.25; 28.42
IV	Both	0.46	0.38; 0.54	−0.04	−2.82; 2.81	0.81	0.71; 0.9	0.72	−1.29; 2.78	0.20	0.15; 0.25	19.38	6.23; 34.17
Rectum													
Overall	Males	6.24	5.84; 6.64	6.66	4.65; 8.7	0.97	0.82; 1.12	−0.2	−2.38; 2.03	0.14	0.08; 0.19	15.45	6.1; 25.62
	Females	4.83	4.47; 5.19	6.5	4.62; 8.42	0.60	0.49; 0.72	−0.07	−3.97; 4	0.10	0.05; 0.15	17.76	6.91; 29.7
<55 years	Males	2.95	2.61; 3.3	7.23	3.94; 10.63	0.26	0.16; 0.37	−2.52	−6.28; 1.4	0.03	0; 0.06	5.68	−3.01; 15.14
	Females	2.90	2.55; 3.25	7.71	5.29; 10.18	0.23	0.13; 0.33	−0.37	−7.52; 7.33	0.07	0.01; 0.12	7.6	−3.91; 20.48
≥55 years	Males	17.01	15.72; 18.3	6.23	4.43; 8.05	3.28	2.74; 3.83	0.33	−2.64; 3.39	0.51	0.3; 0.73	15.28	5.43; 26.06
	Females	10.84	9.82; 11.85	5.69	3.42; 8.01	1.87	1.47; 2.26	−0.2	−4.43; 4.22	0.20	0.07; 0.33	9.29	−1.16; 20.84
*UICC*													
I/II	Both	4.83	4.57; 5.1	7.12	4.79; 9.51	0.15	0.1; 0.2	−5.28	−9.44; −0.93	0.03	0.01; 0.06	12.18	−0.92; 27
III	Both	0.38	0.29; 0.48	3.08	−1.27; 7.61	0.19	0.14; 0.24	0.77	−3.45; 5.18	0.02	0.005; 0.04	7.41	−15.63; 36.75
IV	Both	0.29	0.21; 0.36	4.95	0.05; 10.08	0.44	0.36; 0.51	1.79	−2.1; 5.83	0.06	0.03; 0.08	16.71	4.82; 29.94

Abbreviations: 95%‐CI, 95%‐Confidence interval; APC, annual percentage change between the years 2009–2021; ASR, age‐standardized incidence rates between the years 2019–2021 (cases per million person‐years using the European Standard [1976]); UICC, stage according to union for international cancer control.

**FIGURE 2:**
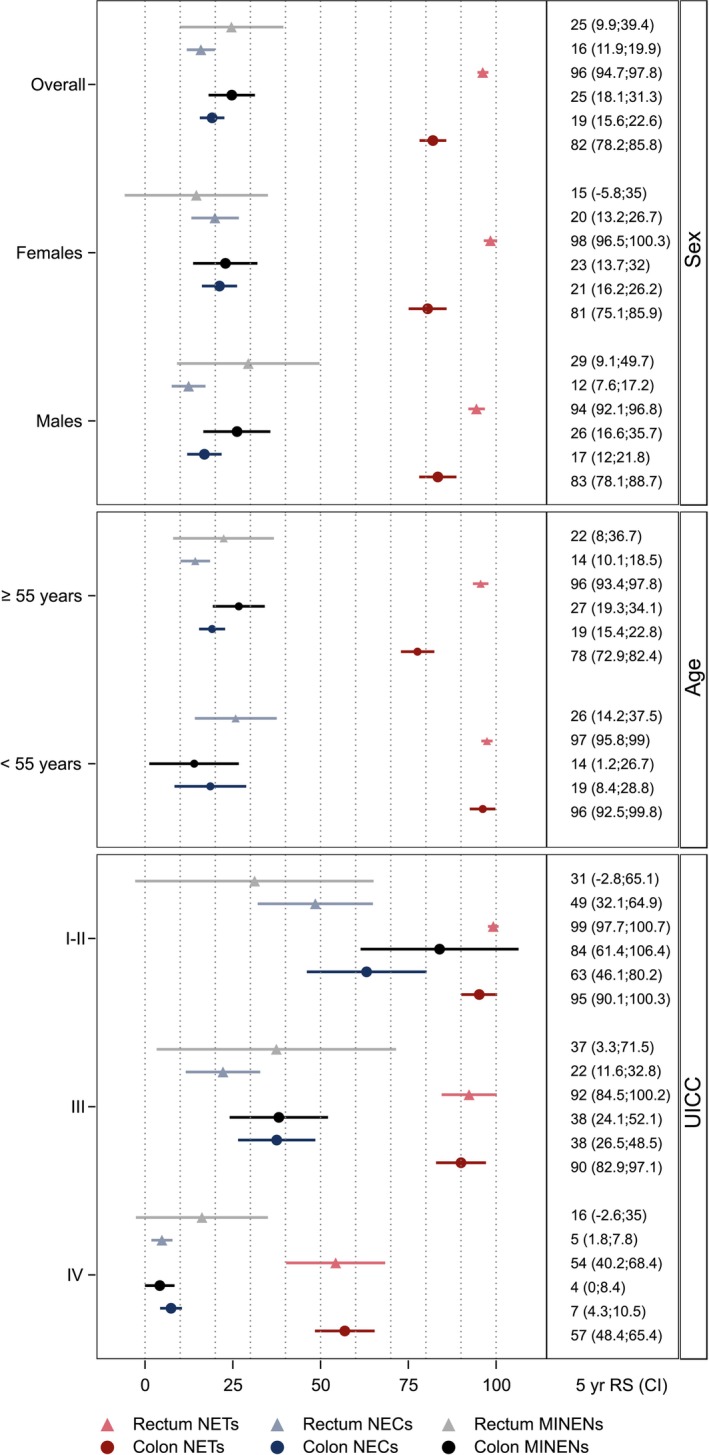
Relative 5‐year survival probability in percent with 95%‐confidence interval for colorectal neuroendocrine neoplasms, period 2017 to 2021, Germany. NEC, poorly differentiated neuroendocrine carcinoma; NET, well‐differentiated neuroendocrine tumor; MiNEN, mixed neuroendocrine‐non‐neuroendocrine neoplasm; UICC, stage according to union for international cancer control.

Figure 2 shows the 5‐year RS of NECs, NETs and MiNENs for the latest period stratified by sex, age groups and staging. Overall, rectal NETs have the highest 5‐year RS (95.9%, 95%‐CI 94.6; 97.1) whereas rectal NECs and colonic MiNENs have the lowest (19.2%, 95%‐CI 15.7; 22.6 and 25.6%, 95%‐CI 20.4; 30.8, respectively). The impact of age on 5‐year RS varied depending on primary site and on morphological subgroup. The difference in survival between younger (<55 years) and older patients was most pronounced for colonic NETs (94.6%, 95%‐CI 91.2; 98.0 vs. 77.4%, 95%‐CI 73.5; 81.2) and rectal NECs (33.4%, 95%‐CI 23.7; 43.1 vs. 16.5%, 95%‐CI 12.8; 20.2). Females have a better 5‐year RS for colonic and rectal NECs than males (23.5%, 95%‐CI 19.2; 27.8 vs. 17.5, 95%‐CI 13.6; 21.4 and 25.3%, 95%‐CI 19.0; 31.6 vs. 15.3, 95%‐CI 11.3; 19.3). Stage had a strong influence on survival of NECs and MiNENs with better prognosis for low stages. In contrast, the survival probabilities for NET stages I–III were similar and indicated a good prognosis, whereas stage IV was associated with a considerably poorer prognosis. In a sensitivity analysis, we excluded a few cancer registries that did not completely close the follow‐up until data extraction. Results of this sensitivity analysis showed that estimates of relative survival only barely changed (<3 percentage points, results not shown).

## 4 DISCUSSION

We assessed the incidence and survival of colorectal NENs according to their primary sites (colon or rectum) and morphological subgroups (NETs, NECs, or MiNENs) within the German population based on cancer registry data. In our study, more NENs were diagnosed in the rectum than in the colon (59% vs. 41%). NETs and NECs were equally frequent in the colon (45% and 44%, respectively), while NET was the predominant morphological subgroup in the rectum (80%). The UICC stage distribution varied depending on the primary site and morphological subgroup: NECs and colonic localization were more likely to be diagnosed at stage IV, while a higher proportion of early stages was observed in association with rectal site and NETs.

We observed an overall increase in the incidence of NETs over time for both sexes. The increase was more pronounced for rectal topography, among younger patients and for UICC I–II. In contrast, we found a constant incidence of NECs in the colon and rectum for both sexes, except for a decrease in the incidence of rectal UICC I–II NECs. The incidence of MiNEN increased markedly except for younger patients and colonic topography.

There were marked differences in survival probability according to primary site and morphology. NETs had the highest RS with a favorable prognosis even at UICC III. Patients with NECs had the lowest RS, and the survival with MiNENs was only slightly higher. Rectal site was associated with increased survival for NETs, while localization did not have a major impact on the overall RS for NECs and MiNENs. Men had a lower survival probability for NECs than women.

The majority of NETs were of grade 1 (see ICD‐O‐3 code 8240 in Table S1) and had lower UICC stages at diagnosis, explaining the clearly better prognosis of NETs compared to other morphological subgroups. MiNENs consist of a poorly differentiated NEC component, together with an adenocarcinoma component. The unfavorable prognosis of patients with MiNENs found in our study is in line with previous literature indicating that the prognosis for MiNEN is worse than for adenocarcinoma.^4^, ^21^

Similar to our findings, previous studies reported a higher occurrence of NETs than NECs in the rectum.^15^, ^39^ The proportion of NEN in the colon is more controversial; Volante et al. observed more NECs than NETs, while a population‐based epidemiological study in the UK suggested a higher frequency of NETs in the colon.^39^, ^40^ In our analysis about one third of the colonic NETs had a distant metastatic spread at the time of diagnosis, which is comparable to previous findings,^40^ however, the high proportion of missing staging information might have biased our results.

A study using surveillance, epidemiology, and end results (SEER) data observed a much higher increase in the incidence of NENs in the rectum than in the colon between 1973 and 2012.^2^ In England, an absolute increase of the age‐standardized incidence of 287% for colonic NENs and of 725% for rectal NENs was observed between 1995 and 2018.^15^ Neither of these studies, however, made a distinction between NETs, NECs and MiNENs. In accordance with our findings, a Norwegian study reported a strong increase in NETs in the colon and rectum between 1993–1996 and 2017–2021, while NECs remained almost constant.^29^ Endoscopy and colorectal cancer screening are regarded as potential contributors to the increase of NETs.^10^, ^12^ An analysis of Dutch screening data found an increase in the incidence of colorectal NETs between 2006 and 2011, followed by a stable incidence until 2016.^41^ The authors suggested the raised awareness of colorectal diseases and a growth in the number of colonoscopies performed due to better access to the healthcare system as potential causes for the increase.

It is challenging to estimate the effect of endoscopies and colorectal cancer screening based on our data as there is hardly any cancer registry data available for the period prior to the introduction of the opportunistic colorectal cancer screening in Germany in 2002. However, a study comparing data from the former East German cancer registry (Nationales Krebsregister der DDR) with its successor the Joint Cancer Registry (GKR) after German reunification provided evidence of the effect.^22^ The particularities of German history, with two German states from 1949 to 1989, allowed to study epidemiological changes in NENs in Germany in the light of two healthcare systems. A relative increase in incidence of 433% in the colon and 2400% in the rectum was observed between the periods 1976–1978 and 2004–2006. The author assumed that the sharp increase in the incidence of NENs is partly due to the general availability of endoscopy after the German reunification.

The increase in incidence of NETs in the younger age groups <55 years cannot be attributed to colorectal cancer screening. During the study period, the FOBT was available for early detection in 50 to 54‐year‐olds, but colonoscopy has only been available for early detection in men aged 50 and over since 2019. Nevertheless, an online survey in 2016 found that 21% of 40‐ to 44‐year‐olds, 28% of 45‐ to 49‐year‐olds and 35% of 50‐ to 54‐year‐olds had a colonoscopy in Germany.^42^ The background of the increasing incidence of NETs in the younger age group requires further investigation. Screening may have also contributed to the increased survival probability by detecting earlier stages; we observed a partial shift towards earlier stages at diagnosis as the incidence of stage I–II NET tumors increased more than stage III–IV tumors.

Further possible reasons for the increase in the incidence besides more frequent use of medical diagnostics are changes in classification standards. For example, the morphology code 8240/3 was listed as Carcinoid tumor, NOS in the first edition of ICD‐O3^43^ and was expanded in the first revision in 2011 to include the terms Neuroendocrine tumor, grade 1; Neuroendocrine carcinoma, low grade; Neuroendocrine carcinoma, well‐differentiated, that is, several equivalent terms have been subsumed under one morphology code. The code 8240/3 accounts for almost half of all NENs in our analysis, and the expansion of the terms may have contributed to a higher diagnosis frequency. Mixed neuroendocrine neoplasms were introduced by the WHO in 2010 as MANEC (mixed adenoneuroendocrine carcinoma) and renamed MiNEN in 2017 allowing for a broader definition to include non‐neuroendocrine parts other than adenocarcinomas.^4^ We attribute the sharp increase in incidence to the recent introduction of MiNEN.

An analysis with data from SEER from 2000 to 2017 using Kaplan–Meier approach showed a higher cancer‐specific survival probability for NECs and MiNENs of the colon and rectum^14^ compared to our results, however, survival was calculated using a different approach. An English study from the National Cancer Registration and Analysis Service (NCRAS) observed an absolute 5‐year survival probability of 90% and 57% for rectal and colonic NETs and 11% and 13% for NECs, respectively.^15^ These results are comparable to the absolute survival probabilities in our cohort (Table S4). Although studies reported improvement of survival in NENs over time, the survival of NECs and MiNENs remained very low, which is confirmed by our results.^2^, ^15^ It is precisely the poor prognosis and high mortality rate that made extrapulmonary NECs a priority for research groups investigating new treatments, despite their rare occurrence.^19^

This is the first and one of the largest studies to analyze colorectal NENs with a particular focus on MiNENs using German cancer registry data. We used data from all 16 German population‐based cancer registries over a 13‐year period. Due to the size of the sample consisting of 12,602 cases, we were able to categorize the cohort by primary site, morphology, and age. The detailed specification of the morphology in the dataset allowed a comprehensive inclusion of all morphological codes described in the literature as colorectal NENs.^4^, ^27^, ^28^, ^29^

One of the limitations is that the period of interest does not cover the incidence before and immediately after the introduction of colorectal cancer screening in 2002. In addition, the cancer registry dataset does not include information on whether a tumor diagnosis was prompted by screening or by a clinical examination following symptoms onset. It is therefore not possible to evaluate the direct effect of screening colonoscopy. During the SARS‐CoV‐2 pandemic in Germany from January 2020, there was a decline in the number of all cancer cases recorded by the cancer registries.^44^ For malignant neoplasms of the colon and rectum, a decrease of 10.6% was observed in 2020 compared to pre‐pandemic values and the incidence remained at a low level in 2021.^44^ No major shifts in the distribution of stages during these years have been observed to date.^45^ The impact of the SARS‐CoV‐2 pandemic on the incidence of colorectal NENs cannot be fully assessed yet, as case documentation for the years 2020 and 2021 is still delayed due to the pandemic.^44^ The missing information on staging is a known challenge with cancer registry data. Rectal NETs had the highest proportion of missing staging data (74%), and the imputed results suggested that the majority of these tumors were probably stage I/II. We assume that the lymph node status was not determined in most rectal NETs with UICC I/II, because rectal T1/G1‐NETs <10 mm have a very low risk of lymph node or distant metastases after endoscopic removal.^18^

We utilized multiple imputation to treat the high proportion of missing UICC values. A correctly specified multiple imputation model yields unbiased estimates under the assumption of missingness at random, which is a plausible scenario if a set of relevant predictor for missingness exists.^46^ However, if missingness is not at random, the imputed results may be still biased. In a recent record linkage study, multiple imputation delivered good estimates of the missing true staging values even with a very limited number of predictors, suggesting the robustness of the method in cancer registry data.^47^ Due to the lack of similar studies addressing the issue, it is difficult to further assess the risk and extent of bias potentially introduced by the substantial proportion of missing values.

Our registry‐based study suggests that the incidence of both colonic and rectal NETs increased within the German population between 2009 and 2021. The growing availability of colonoscopy for screening and for diagnostic purposes could have contributed to this increase especially among older patients. However, the increasing incidence of NETs among younger patients warrants further inspection of other factors influencing the incidence of colorectal NENs.

Even if UICC stage has a major influence on the probability of survival, the background of the large discrepancies in survival probability of NENs with different primary site and morphology should be investigated more in depth.

## AUTHOR CONTRIBUTIONS

**Lennart Möller:** Conceptualization; investigation; writing – original draft; data curation; formal analysis; visualization; methodology. **Andras Szentkirályi:** Writing – review and editing; formal analysis. **Christine Eisfeld:** Validation; conceptualization; writing – review and editing. **Ina Wellmann:** Validation; visualization; writing – review and editing. **Franziska Rees:** Resources; writing – review and editing. **Kevin Claaßen:** Writing – review and editing. **Florian Oesterling:** Writing – review and editing. **Hiltraud Kajüter:** Conceptualization; writing – review and editing; supervision. **Andreas Stang:** Conceptualization; writing – review and editing; supervision.

## CONFLICT OF INTEREST STATEMENT

The authors declare no conflicts of interest.

## Supporting information


**DATA S1.** Supporting information.

## Data Availability

The data underlying this article can be obtained from the Centre for Cancer Registry Data (ZfKD) on reasoned request. Further information is available from the corresponding author upon request.

## References

[1] Rindi G , Klimstra DS , Abedi‐Ardekani B , et al. A common classification framework for neuroendocrine neoplasms: an International Agency for Research on Cancer (IARC) and World Health Organization (WHO) expert consensus proposal. Mod Pathol. 2018;31(12):1770‐1786. doi:10.1038/s41379-018-0110-y 30140036 PMC6265262

[2] Dasari A , Shen C , Halperin D , et al. Trends in the incidence, prevalence, and survival outcomes in patients with neuroendocrine tumors in the United States. JAMA Oncol. 2017;3(10):1335. doi:10.1001/jamaoncol.2017.0589 28448665 PMC5824320

[3] Dasari A , Mehta K , Byers LA , Sorbye H , Yao JC . Comparative study of lung and extrapulmonary poorly differentiated neuroendocrine carcinomas: a SEER database analysis of 162,983 cases. Cancer. 2018;124(4):807‐815. doi:10.1002/cncr.31124 29211313 PMC5801102

[4] WHO Classification of Tumours Editorial Board . Digestive System Tumours [Internet]. Vol 1. 5th ed. International Agency for Research on Cancer; 2019.

[5] Nagtegaal ID , Odze RD , Klimstra D , et al. The 2019 WHO classification of tumours of the digestive system. Histopathology. 2020;76(2):182‐188. doi:10.1111/his.13975 31433515 PMC7003895

[6] Caplin M , Sundin A , Nillson O , et al. ENETS consensus guidelines for the Management of Patients with digestive neuroendocrine neoplasms: colorectal neuroendocrine neoplasms. Neuroendocrinology. 2012;95(2):88‐97. doi:10.1159/000335594 22261972

[7] Ramage JK , de Herder WW , Delle Fave G , et al. ENETS consensus guidelines update for colorectal neuroendocrine neoplasms. Neuroendocrinology. 2016;103(2):139‐143. doi:10.1159/000443166 26730835

[8] Leoncini E , Boffetta P , Shafir M , Aleksovska K , Boccia S , Rindi G . Increased incidence trend of low‐grade and high‐grade neuroendocrine neoplasms. Endocrine. 2017;58(2):368‐379. doi:10.1007/s12020-017-1273-x 28303513 PMC5671554

[9] Fraenkel M , Kim M , Faggiano A , de Herder WW , Valk GD . __Incidence of gastroenteropancreatic neuroendocrine tumours: a systematic review of the literature. Endocr Relat Cancer. 2014;21(3):R153‐R163. doi:10.1530/ERC-13-0125 24322304

[10] Cope J , Srirajaskanthan R . Rectal neuroendocrine neoplasms: why is there a global variation? Curr Oncol Rep. 2022;24(3):257‐263. doi:10.1007/s11912-021-01172-1 35084662 PMC8885478

[11] Leoncini E , Carioli G , La Vecchia C , Boccia S , Rindi G . Risk factors for neuroendocrine neoplasms: a systematic review and meta‐analysis. Ann Oncol. 2016;27(1):68‐81. doi:10.1093/annonc/mdv505 26487581

[12] Yao JC , Hassan M , Phan A , et al. One hundred years after “carcinoid”: epidemiology of and prognostic factors for neuroendocrine tumors in 35,825 cases in the United States. J Clin Oncol. 2008;26(18):3063‐3072. doi:10.1200/JCO.2007.15.4377 18565894

[13] Mangiapane S , Kretschmann J , Czihal T , von Stillfried D . Veränderung der vertragsärztlichen Leistungsinanspruchnahme während der COVID‐Krise. Tabellarischer Trendreport bis zum 1. Halbjahr 2022. Zentralinstitut für die Kassenärztliche Versorgung in der Bundesrepublik Deutschland; 2022.

[14] Keller HR , Senapathi SH , Morada A , Bertsch D , Cagir B . Survival in patients with neuroendocrine tumors of the colon, rectum and small intestine. Am J Surg. 2023;225(1):58‐65. doi:10.1016/j.amjsurg.2022.09.053 36216612

[15] White BE , Rous B , Chandrakumaran K , et al. Incidence and survival of neuroendocrine neoplasia in England 1995–2018: a retrospective, population‐based study. Lancet Reg Health: Eur. 2022;23:100510. doi:10.1016/j.lanepe.2022.100510 36176500 PMC9513765

[16] Man D , Wu J , Shen Z , Zhu X . Prognosis of patients with neuroendocrine tumor: a SEER database analysis. Cancer Manag Res. 2018;10:5629‐5638. doi:10.2147/CMAR.S174907 30519109 PMC6239108

[17] Tsoli M , Chatzellis E , Koumarianou A , Kolomodi D , Kaltsas G . Current best practice in the management of neuroendocrine tumors. Ther Adv Endocrinol Metab. 2019;10:204201881880469. doi:10.1177/2042018818804698 PMC6378464 30800264

[18] Rinke A , Ambrosini V , Dromain C , et al. European neuroendocrine tumor society (enets) 2023 guidance paper for colorectal neuroendocrine tumours. J Neuroendocrinol. 2023;35(6):e13309. doi:10.1111/jne.13309 37345509

[19] Robinson MD , Livesey D , Hubner RA , Valle JW , McNamara MG . Future therapeutic strategies in the treatment of extrapulmonary neuroendocrine carcinoma: a review. Ther Adv Med Oncol. 2023;15:175883592311568. doi:10.1177/17588359231156870 PMC9983111 36872945

[20] Garcia‐Carbonero R , Sorbye H , Baudin E , et al. ENETS consensus guidelines for high‐grade Gastroenteropancreatic neuroendocrine tumors and neuroendocrine carcinomas. Neuroendocrinology. 2016;103(2):186‐194. doi:10.1159/000443172 26731334

[21] Sorbye H , Grande E , Pavel M , et al. European neuroendocrine tumor society (ENETS) 2023 guidance paper for digestive neuroendocrine carcinoma. J Neuroendocrinol. 2023;35(3):e13249. doi:10.1111/jne.13249 36924180

[22] Scherübl H , Brigitte S , Roland S , et al. Clinically detected gastroenteropancreatic neuroendocrine tumors are on the rise: epidemiological changes in Germany. World J Gastroenterol. 2013;19(47):9012. doi:10.3748/wjg.v19.i47.9012 24379626 PMC3870554

[23] Grundmann N , Voigtländer S , Hakimhashemi A , Pape U , Meyer M , Müller‐Nordhorn J . Site‐specific trends in gastroenteropancreatic neuroendocrine neoplasms in Bavaria, Germany. Cancer Med. 2023;12(19):19949‐19958. doi:10.1002/cam4.6510 37737059 PMC10587981

[24] Zentrum Für Krebsregisterdaten (ZfKD) Im Robert Koch‐Institut . Datensatz des ZfKD auf Basis der epidemiologischen Landeskrebsregisterdaten Epi2022_2, verfügbare Diagnosejahre bis 2021. 2024. doi:10.18444/5.03.01.0005.0018.0002

[25] Levine EA , Blazer DG , Kim MK , et al. Gene expression profiling of peritoneal metastases from appendiceal and colon cancer demonstrates unique biologic signatures and predicts patient outcomes. J Am Coll Surg. 2012;214(4):599‐606. doi:10.1016/j.jamcollsurg.2011.12.028 22342786 PMC3768122

[26] Moris D , Diamantis TI , Vagios S , et al. Neuroendocrine neoplasms of the appendix: a review of the literature. Anticancer Res. 2018;38(2):601‐612. doi:10.21873/anticanres.12264 29374682

[27] World Health Organization . International Classification of Diseases for Oncology (ICD‐O), 2nd revision. 3rd ed. World Health Organization; 2019.

[28] WHO Classification of Tumours Editorial Board . Endocrine and Neuroendocrine Tumours [Internet]. Vol 10. 5th ed. International Agency for Research on Cancer; 2022.

[29] Thiis‐Evensen E , Boyar Cetinkaya R . Incidence and prevalence of neuroendocrine neoplasms in Norway 1993–2021. J Neuroendocrinol. 2023;35(4):13264. doi:10.1111/jne.13264 36988112

[30] Brierley JD , Gospodarowicz MK , Wittekind C , Brierley JD . TNM Classification of Malignant Tumours. 8th ed. John Wiley & Sons, Incorporated; 2017.

[31] Doll R , Cook P . Summarizing indices for comparison of cancer incidence data. Int J Cancer. 1967;2(3):269‐279. doi:10.1002/ijc.2910020310 6041994

[32] Berkson J , Gage RP . Calculation of survival rates for cancer. Proc Staff Meet Mayo Clin. 1950;25(11):270‐286. 15417650

[33] Ederer F , Axtell LM , Cutler SJ . The relative survival rate: a statistical methodology. Natl Cancer Inst Monogr. 1961;6:101‐121. 13889176

[34] Hakulinen T , Seppä K , Lambert PC . Choosing the relative survival method for cancer survival estimation. Eur J Cancer. 2011;47(14):2202‐2210. doi:10.1016/j.ejca.2011.03.011 21549589

[35] Brenner H , Gefeller O . An alternative approach to monitoring cancer patient survival. Cancer. 1996;78(9):2004‐2010. 8909323

[36] Brenner H , Gefeller O , Hakulinen T . A computer program for period analysis of cancer patient survival. Eur J Cancer. 2002;38(5):690‐695. doi:10.1016/S0959-8049(02)00003-5 11916552

[37] R Core Team . R: A Language and Environment for Statistical Computing. R Foundation for Statistical Computing, Vienna, Austria. 2023 https://www.R-project.org/

[38] Holleczek B , Gondos A , Brenner H . periodR: an R package to calculate long‐term cancer survival estimates using period analysis. Methods Inf Med. 2009;48(2):123‐128. doi:10.3414/ME0563 19283308

[39] Genus TSE , Bouvier C , Wong KF , et al. Impact of neuroendocrine morphology on cancer outcomes and stage at diagnosis: a UK nationwide cohort study 2013–2015. Br J Cancer. 2019;121(11):966‐972. doi:10.1038/s41416-019-0606-3 31649320 PMC6889414

[40] Volante M , Grillo F , Massa F , et al. Neuroendocrine neoplasms of the appendix, colon and rectum. Pathologica. 2021;113(1):19‐27. doi:10.32074/1591-951X-230 33686307 PMC8138694

[41] Kooyker AI , Verbeek WH , Van Den Berg JG , Tesselaar ME , Van Leerdam ME . Change in incidence, characteristics and management of colorectal neuroendocrine tumours in The Netherlands in the last decade. United Eur Gastroenterol J. 2020;8(1):59‐67. doi:10.1177/2050640619865113 PMC7006007 32213058

[42] Weigl K , Tikk K , Hoffmeister M , et al. Prevalence of a first‐degree relative with colorectal cancer and uptake of screening among persons 40 to 54 years old. Clin Gastroenterol Hepatol. 2020;18(11):2535‐2543.e3. doi:10.1016/j.cgh.2019.11.044 31809916

[43] Fritz AG , ed. International Classification of Diseases for Oncology: ICD‐O. 3rd ed. World Health Organization; 2000.

[44] Kraywinkel K , Imhoff M , Voigtländer S , Stang A . Krebsneuerkrankungen in Pandemiezeiten: Ergebnisse aus deutschen Krebsregistern für das Diagnosejahr 2020. Onkol. 2024;30(4):265‐272. doi:10.1007/s00761-023-01444-4

[45] Brenner H , Cardoso R , Heisser T , Hoffmeister M , Holleczek B . Indications of substantial delay of colorectal cancer diagnoses due to COVID‐19 in Germany. Lancet reg Health Eur. 2022;23:100543. doi:10.1016/j.lanepe.2022.100543 36381140 PMC9646477

[46] Sterne JAC , White IR , Carlin JB , et al. Multiple imputation for missing data in epidemiological and clinical research: potential and pitfalls. BMJ. 2009;338:b2393. doi:10.1136/bmj.b2393 19564179 PMC2714692

[47] Luo Q , Egger S , Yu XQ , Smith DP , O'Connell DL . Validity of using multiple imputation for “unknown” stage at diagnosis in population‐based cancer registry data. PLoS One. 2017;12(6):e0180033. doi:10.1371/journal.pone.0180033 28654653 PMC5487067

