# Supplementary material for: Incidence trends and relative survival of colorectal neuroendocrine neoplasms: A population‐based study using German cancer registry data
Source: Int J Cancer. 2025 Feb 20;157(1):116–25. doi: 10.1002/ijc.35372 (PMC12062926; doi:10.1002/ijc.35372)
Supplement: Supplementary file 1 — DATA S1. Supporting information. [file IJC-157-116-s001.pdf]

Supplementary Material

Incidence trends and relative survival of colorectal neuroendocrine neoplasms - A population-based study using German cancer registry data

Lennart Möller, Andras Szentkirályi, Christine Eisfeld, Ina Wellmann, Franziska Rees, Kevin Claaßen, Florian Oesterling, Hiltraud Kajüter, Andreas Stang

Table of Contents:

Supplementary materials & methods: 2

Supplement Figure 1 Age-specific incidence rates for colorectal neuroendocrine neoplasms, 2009-2021, Germany........ 3

Supplement Table 1 Distribution of morphologies and grading of neuroendocrine neoplasms (NENs) with colorectal primary site according to ICD-O-3, 2009-2021, Germany. 4

Supplement Table 2 Age-standardized incidence rates per million person-years (world standard population) for colorectal neuroendocrine neoplasms, 2019-2021, Germany. 5

Supplement Table 3 Age-standardized incidence rates per million person-years (U.S. 2000 standard population) for colorectal neuroendocrine neoplasms, 2019-2021, Germany. 6

Supplement Table 4 Absolute 5-year survival probability in percent for colorectal neuroendocrine neoplasms, period 2017 to 2021, Germany. 7

Supplement Figure 2 Frequencies of imputed categorical variables in the original vs imputed datasets........ 8

Supplement Figure 3 Age-standardized incidence rates for colorectal neuroendocrine neoplasms stratified by UICC, 2009-2021, Germany. 9

Supplementary references........ 10

Supplementary materials & methods

Imputation model

Multiple imputation by chained equations (MICE) assuming missingness at random was performed to handle the high proportion of missing values of UICC stages (see table 1). In the first step, the missing T-, N-, and M-stages were imputed using age at diagnosis, sex, year of diagnosis, status of morphological verification, grading, anatomic subsite, morphology group (NET, NEC, or MINEN), vital status at the end of the period (i.e. alive or deceased), and cumulative baseline hazard as predictor variables. T-, N-, and M-stages were also included as predictors, because they were associated with each other and their missing patterns were not completely overlapping. The imputation method for T- and N-values was polytomous regression, while M-stages were imputed by logistic regression. The Nelson-Aalen estimator was used to approximate the cumulative baseline hazard[1]. The number of imputed datasets was set to 100, and the number of maximum iterations was 20. Convergence was diagnosed by plotting the mean and standard deviation of each imputed variable against the iteration number for each parallel stream. The UICC-stages were derived from the imputed TNM-values in a subsequent step (passive imputation)[2]. Age-standardized incidence rates, annual percentage changes and relative 5-year survival were calculated for each imputed dataset stratified by UICC stages, morphology groups and primary site. As the estimates were normally distributed across the imputed datasets, the untransformed outcomes were pooled using the mean value as a point estimate and the standard error was obtained according to Rubin's rule[3]. The imputation was performed with R 4.3.2[4] using the mice package (version 3.16.0) [5].

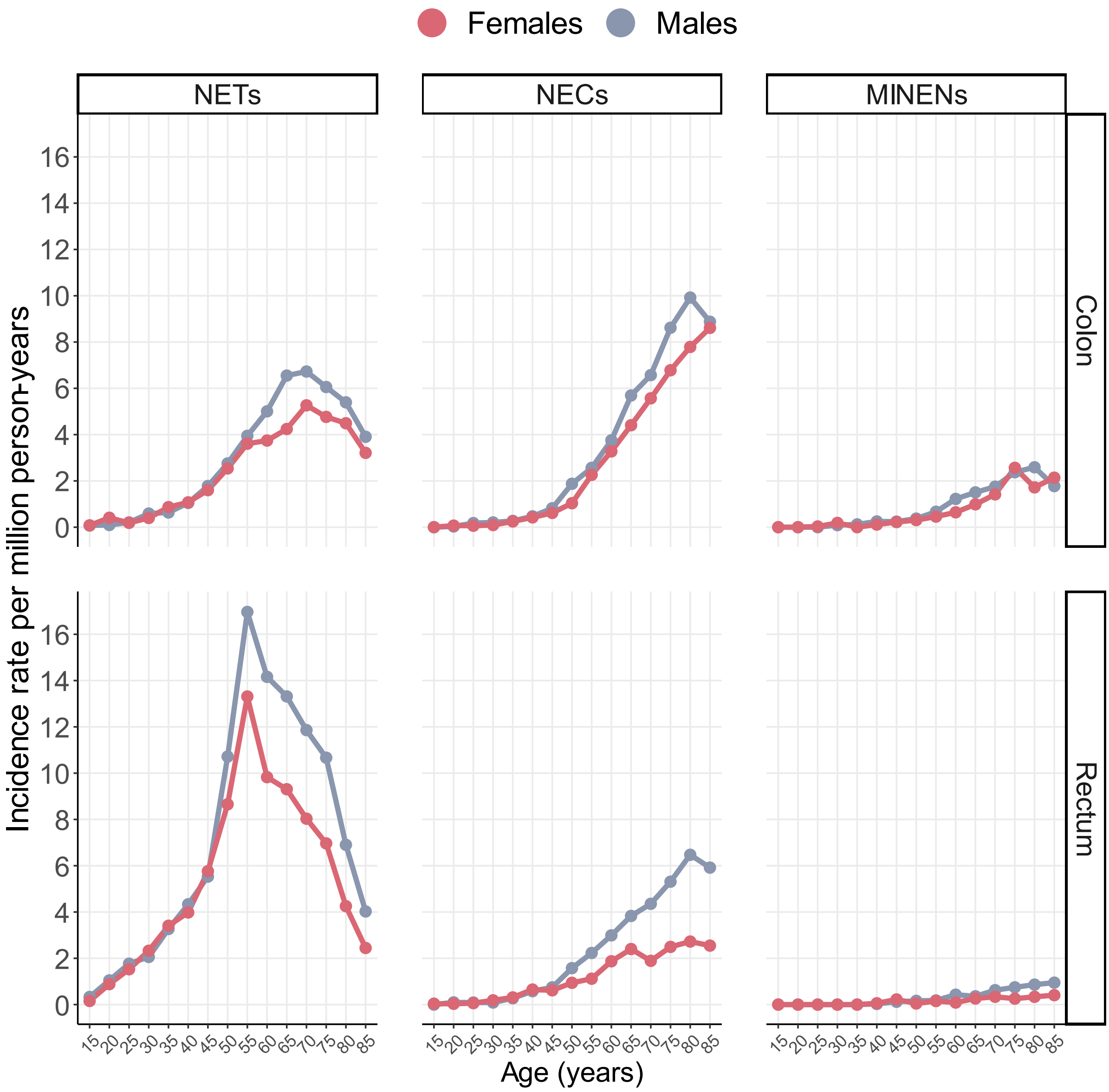

Supplement Figure 1 Age-specific incidence rates for colorectal neuroendocrine neoplasms, 2009-2021, Germany**.**

Legend:

NET: Well-differentiated Neuroendocrine Tumor

NEC: Poorly differentiated Neuroendocrine Carcinoma

MiNEN: Mixed neuroendocrine-non-neuroendocrine Neoplasm

Supplement Table 1 Distribution of morphologies and grading of neuroendocrine neoplasms (NENs) with colorectal primary site according to ICD-O-3, 2009-2021, Germany.

NEN	Code and term	n (%)	Grading, %		
			G1	G2	G3
NET	8152/3 - L-cell tumor	2 (0)	50	50	0
NET	8240/3 - Neuroendocrine tumor, NOS	7241 (57.5)	92.6	5.2	2.2
NET	8241/3 - Enterochromaffin cell carcinoid	28 (0.2)	88.5	0	11.5
NET	8249/3 - Neuroendocrine tumor, grade 2	1047 (8.3)	3.9	91.2	4.9
NEC	8013/3 - Large cell neuroendocrine carcinoma	584 (4.6)	1	2.9	96.2
NEC	8041/3 - Small cell carcinoma, NOS	529 (4.2)	1	6.9	92.1
NEC	8246/3 - Neuroendocrine carcinoma, NOS[a,b]	2423 (19.2)	0	0	100
MiNEN	8244/3 - Mixed adenoneuroendocrine carcinoma	684 (5.4)	1.9	12	86.1
MiNEN	8245/3 - Adenocarcinoid tumor	8 (0.1)	42.9	42.9	14.3
MiNEN	8154/3 - Mixed neuroendocrine non-neuroendocrine neoplasm (MiNEN)	56 (0.4)	1.9	9.6	88.5

[a]950 cases originally coded as 8246 with grade 1 had been recoded as 8240

[b]295 cases originally coded as 8246 with grade 2 had been recoded as 8249

Supplement Table 2 Age-standardized incidence rates per million person-years (world standard population) for colorectal neuroendocrine neoplasms, 2019-2021, Germany.

		Well-differentiated Neuroendocrine Tumors (NETs)		Poorly differentiated Neuroendocrine Carcinomas (NECs)		Mixed neuroendocrine-non-neuroendocrine Neoplasm (MiNENs)	
	**Sex**	**Rate**	**95%-CI**	**Rate**	**95%-CI**	**Rate**	**95%-CI**
**Colon**							
Overall	Males	1.36	1.19; 1.52	0.95	0.83; 1.08	0.25	0.19; 0.31
	Females	1.20	1.04; 1.36	0.68	0.58; 0.78	0.25	0.19; 0.31
< 55 Years	Males	0.95	0.78; 1.13	0.36	0.25; 0.47	0.15	0.09; 0.22
	Females	1.01	0.81; 1.2	0.29	0.2; 0.39	0.11	0.05; 0.18
≥ 55 Years	Males	5.47	4.74; 6.21	4.59	3.93; 5.24	1.38	1.01; 1.74
	Females	4.55	3.9; 5.19	3.56	3.02; 4.1	1.29	0.96; 1.61
**UICC**							
I/II	Both	0.59	0.51; 0.68	0.10	0.07; 0.13	0.03	0.02; 0.04
III	Both	0.35	0.28; 0.42	0.16	0.12; 0.2	0.08	0.05; 0.1
IV	Both	0.33	0.27; 0.39	0.56	0.49; 0.63	0.14	0.11; 0.18
**Rectum**							
Overall	Males	4.71	4.39; 5.02	0.68	0.57; 0.79	0.09	0.05; 0.13
	Females	3.73	3.44; 4.02	0.44	0.35; 0.53	0.07	0.03; 0.11
< 55 Years	Males	3.94	3.56; 4.31	0.32	0.22; 0.42	0.05	0.01; 0.09
	Females	3.83	3.46; 4.2	0.29	0.19; 0.39	0.07	0.02; 0.12
≥ 55 Years	Males	17.33	15.99; 18.67	3.20	2.65; 3.75	0.50	0.28; 0.72
	Females	11.19	10.12; 12.25	1.85	1.44; 2.25	0.19	0.06; 0.32
**UICC**							
I/II	Both	3.69	3.48; 3.9	0.11	0.07; 0.15	0.02	0.007; 0.04
III	Both	0.29	0.22; 0.37	0.13	0.09; 0.17	0.02	0.002; 0.03
IV	Both	0.22	0.15; 0.28	0.31	0.25; 0.36	0.04	0.02; 0.06

Legend:

UICC: Stage according to Union for International Cancer Control

Supplement Table 3 Age-standardized incidence rates per million person-years (U.S. 2000 standard population) for colorectal neuroendocrine neoplasms, 2019-2021, Germany.

		**Well-differentiated Neuroendocrine Tumors (NETs)**		**Poorly differentiated Neuroendocrine Carcinomas (NECs)**		**Mixed neuroendocrine-non-neuroendocrine Neoplasm (MiNENs)**	
	**Sex**	**Rate**	**95%-CI**	**Rate**	**95%-CI**	**Rate**	**95%-CI**
**Colon**							
Overall	Males	1.85	1.64; 2.07	1.46	1.28; 1.65	0.38	0.29; 0.47
	Females	1.62	1.42; 1.82	1.11	0.96; 1.25	0.40	0.31; 0.48
< 55 Years	Males	0.76	0.58; 0.95	0.38	0.25; 0.51	0.07	0.02; 0.12
	Females	0.74	0.56; 0.92	0.21	0.11; 0.31	0.08	0.02; 0.14
≥ 55 Years	Males	5.62	4.9; 6.34	5.26	4.56; 5.96	1.53	1.15; 1.91
	Females	4.72	4.11; 5.33	4.32	3.75; 4.9	1.53	1.18; 1.87
**UICC**							
I/II	Both	0.76	0.66; 0.87	0.17	0.12; 0.21	0.05	0.03; 0.07
III	Both	0.49	0.41; 0.58	0.25	0.2; 0.31	0.13	0.09; 0.17
IV	Both	0.47	0.39; 0.55	0.86	0.76; 0.96	0.21	0.16; 0.26
**Rectum**							
Overall	Males	5.91	5.53; 6.3	1.01	0.86; 1.16	0.14	0.09; 0.2
	Females	4.58	4.23; 4.93	0.61	0.49; 0.73	0.10	0.05; 0.16
< 55 Years	Males	2.96	2.61; 3.32	0.27	0.16; 0.38	0.03	0; 0.06
	Females	2.95	2.59; 3.31	0.23	0.13; 0.33	0.08	0.01; 0.14
≥ 55 Years	Males	15.95	14.75; 17.15	3.54	2.97; 4.12	0.56	0.33; 0.79
	Females	9.80	8.9; 10.7	1.95	1.56; 2.33	0.20	0.07; 0.32
**UICC**							
I/II	Both	4.57	4.32; 4.82	0.15	0.1; 0.2	0.04	0.01; 0.06
III	Both	0.37	0.28; 0.46	0.19	0.14; 0.24	0.03	0.007; 0.05
IV	Both	0.27	0.2; 0.35	0.45	0.38; 0.52	0.05	0.03; 0.08

Legend:

UICC: Stage according to Union for International Cancer Control

Supplement Table 4 Absolute 5-year survival probability in percent for colorectal neuroendocrine neoplasms, period 2017 to 2021, Germany.

	Well-differentiated Neuroendocrine Tumors (NETs)		Poorly differentiated Neuroendocrine Carcinomas (NECs)		Mixed neuroendocrine-non-neuroendocrine Neoplasm (MiNENs)	
	**Absolute 5-year survival probability**	**95%- Confidence interval**	**Absolute 5-year survival probability**	**95%- Confidence interval**	**Absolute 5-year survival probability**	**95%- Confidence interval**
**Colon**						
Overall	73.2	70.4; 76	17.1	14.7; 19.5	21.6	17.2; 26
Overall Males	73.3	69.5; 77.2	14.5	11.3; 17.8	20.7	14.7; 26.7
Overall Females	73.1	69; 77.1	19.6	16; 23.2	22.3	15.9; 28.7
<55 Years	93.4	90.1; 96.8	20.7	12.1; 29.2	21	9; 33
>=55Years	67.4	64; 70.8	16.7	14.1; 19.2	21.6	16.9; 26.4
**UICC**						
I-II	86.2	82.2; 90.2	55.8	45.9; 65.7	70.2	54.9; 85.6
III	78.3	73.2; 83.3	27.8	20.7; 34.8	31.6	22.5; 40.7
IV	49.7	43.4; 56.1	6.6	4.4; 8.7	5.6	2.3; 9
**Rectum**						
Overall	89.6	88.4; 90.8	16.7	13.7; 19.8	23.4	14.5; 32.3
Overall Males	87.0	85.2; 88.7	13.3	9.8; 16.8	21.9	11; 32.8
Overall Females	92.8	91.3; 94.3	22.3	16.7; 27.8	24.8	9.6; 39.9
<55 Years	96.8	95.6; 98	33.0	23.4; 42.6	32.1	6.5; 57.7
>=55Years	86.0	84.4; 87.7	13.8	10.7; 16.8	21.4	12.1; 30.6
**UICC**						
I-II	92.7	91.5; 93.9	45.3	34.5; 56.1	51	25.8; 76.2
III	85.3	79.4; 91.1	23.3	15.5; 31.1	27.4	10.7; 44
IV	42.5	32.2; 52.9	5.3	2.9; 7.8	6.9	-1.7; 15.6

Legend:

UICC: Stage according to Union for International Cancer Control

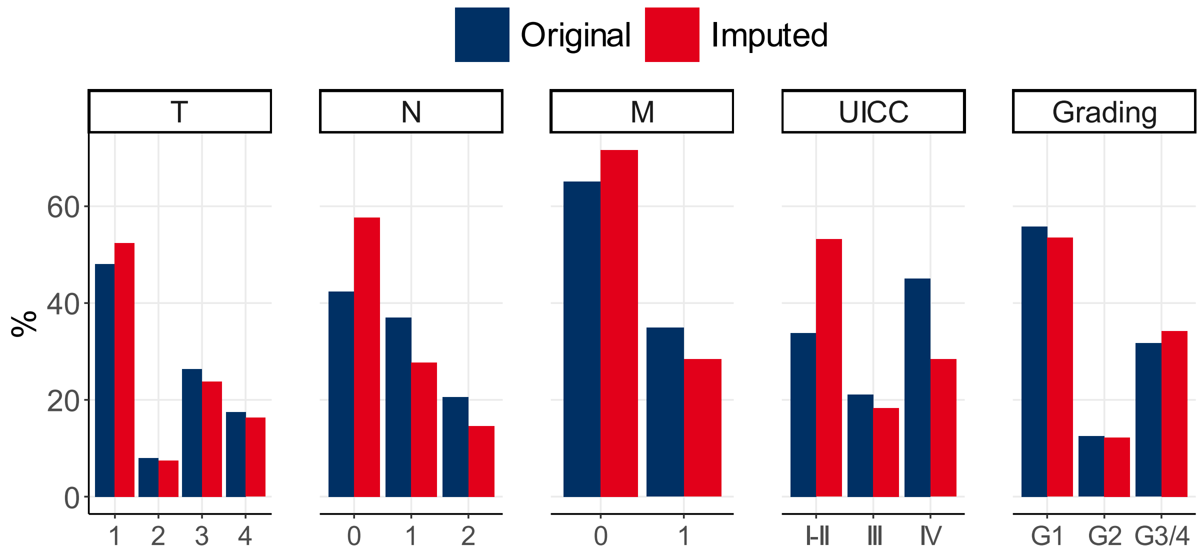

Supplement Figure 2 Frequencies of imputed categorical variables in the original vs imputed datasets.

Legend:
UICC: Stage according to Union for International Cancer Control

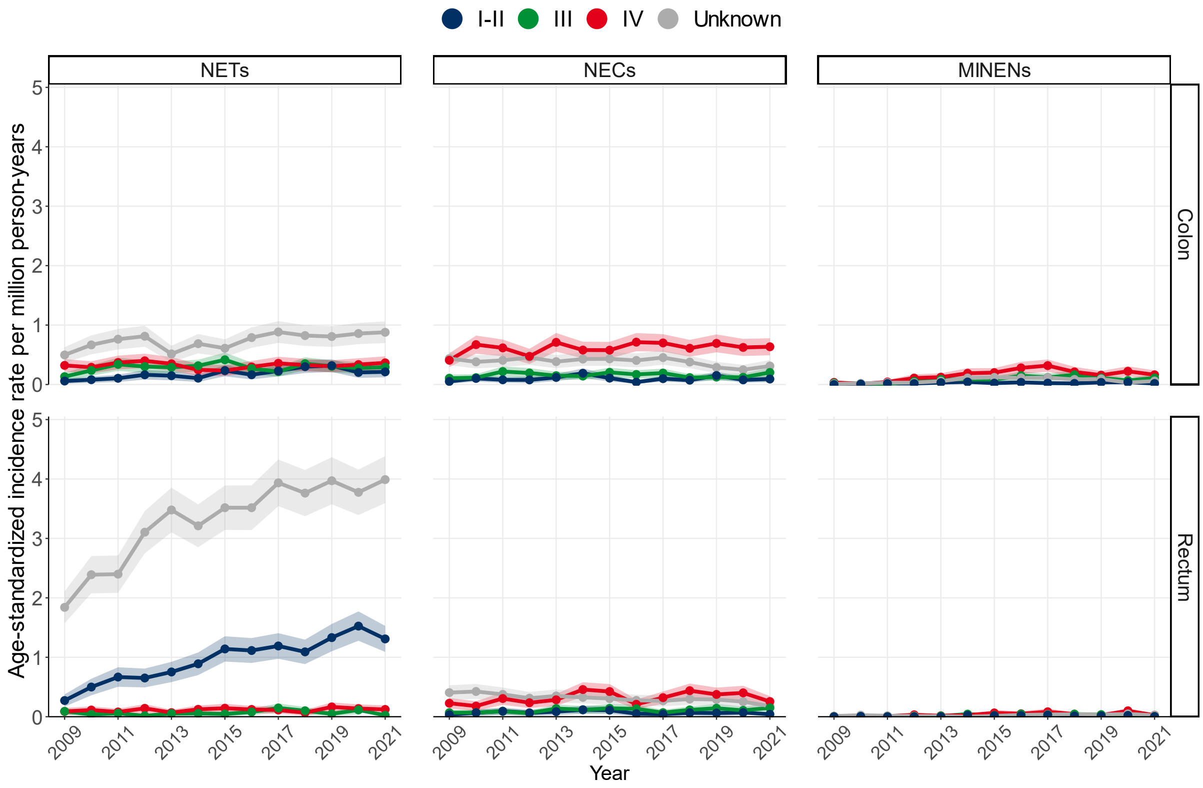

Supplement Figure 3 Age-standardized incidence rates for colorectal neuroendocrine neoplasms stratified by UICC, 2009-2021, Germany.

Legend:

NET: Well-differentiated Neuroendocrine Tumors

NEC: Poorly differentiated Neuroendocrine Carcinomas

MiNEN: Mixed neuroendocrine-non-neuroendocrine Neoplasm

UICC: Stage according to Union for International Cancer Control

Supplementary references

1. White IR, Royston P. Imputing missing covariate values for the Cox model. Stat Med. 2009;28(15):1982-1998. doi:10.1002/sim.3618
2. Van Buuren S. Flexible Imputation of Missing Data, Second Edition. 2nd ed. Chapman and Hall/CRC; 2018. doi:10.1201/9780429492259
3. Rubin DB. Multiple Imputation for Nonresponse in Surveys. 1st ed. Wiley; 1987. doi:10.1002/9780470316696
4. R Core Team. R: A Language and Environment for Statistical Computing. R Foundation for Statistical Computing, Vienna, Austria. https://www.R-project.org/. Published online 2023.
5. Buuren SV, Groothuis-Oudshoorn K. mice: Multivariate Imputation by Chained Equations in R. J Stat Soft. 2011;45(3). doi:10.18637/jss.v045.i03
